# Optimization of codon composition and regulatory elements for expression of human insulin like growth factor-1 in transgenic chloroplasts and evaluation of structural identity and function

**DOI:** 10.1186/1472-6750-9-33

**Published:** 2009-04-03

**Authors:** Henry Daniell, Gricel Ruiz, Bela Denes, Laurence Sandberg, William Langridge

**Affiliations:** 1 Department of Molecular Biology and Microbiology, University of Central Florida, College of Medicine, Biomolecular Science Building, Room 336, Orlando, FL 32816-2364, USA; 2 Center for Health Disparities and Molecular Medicine, 161 Mortensen Hall, Loma Linda University, Loma Linda, CA 92350, USA; 3 Department of Biochemistry, Loma Linda University, Loma Linda, CA, 92350, USA

## Abstract

**Background:**

Transgenic chloroplasts are potential bioreactors for recombinant protein production, especially for achievement of high levels of protein expression and proper folding. Production of therapeutic proteins in leaves provides transgene containment by elimination of reproductive structures. Therefore, in this study, human Insulin like Growth Factor-1 is expressed in transgenic chloroplasts for evaluation of structural identity and function.

**Results:**

Expression of the synthetic Insulin like Growth Factor 1 gene (*IGF-1s*, 60% AT) was observed in transformed *E. coli*. However, no native *IGF-1 *gene (*IGF-1n*, 41% AT) product was detected in the western blots in *E. coli*. Site-specific integration of the transgenes into the tobacco chloroplast genome was confirmed after transformation using PCR. Southern blot analysis confirmed that the transgenic lines were homoplasmic. The transgenic plant lines had *IGF-1s *expression levels of 11.3% of total soluble protein (TSP). The *IGF-1n *plants contained 9.5% TSP as *IGF-1n*, suggesting that the chloroplast translation machinery is more flexible than *E. coli *in codon preference and usage. The expression of *IGF-1 *was increased up to 32% TSP under continuous illumination by the chloroplast light regulatory elements. IgG-Sepharose affinity column chromatographic separation of Z domain containing chloroplast derived IGF-1 protein, single and two dimensional electrophoresis methods and mass spectrometer analysis confirmed the identity of human IGF-1 in transgenic chloroplasts. Two spots analyzed from 2-D focusing/phoresis acrylamide gel showed the correct amino acid sequence of human IGF-1 and the *S. aureus *Z-tag. Cell proliferation assays in human HU-3 cells demonstrated the biological activity of chloroplast derived IGF-1 even in the presence of the *S. aureus *Z tag.

**Conclusion:**

This study demonstrates that the human Insulin like Growth Factor-1 expressed in transgenic chloroplasts is identical to the native protein and is fully functional. The ability to use plant chloroplasts as bioreactors to generate proteins of great economic value that retain their biological activity is an exciting and achievable goal that appears to be within our grasp.

## Background

Insulin-like growth factor 1 is an anabolic hormone produced in the liver that is known to stimulate proliferation and differentiation of many cell types and plays an important role in tissue renewal and repair [1]. Growth hormone binds to specific receptors on the hepatocyte cell membrane and triggers a mechanism (largely undefined), that synthesizes and releases IGF-1 into the blood [2]. The normal levels of IGF-1 are between 120–400 ng/ml [3]. Because of important IGF-1 functions in the body, people who suffer IGF-1 deficiency also experience many harmful side effects [4]. Patients with liver cirrhosis have a reduction of the GH receptor in the hepatocytes and the diminished synthesis of the liver parenchyma causes a significant decrease of IGF-I levels in the blood (20 ng/ml and frequently to undetectable levels). This reduction in IGF-1 results in systemic problems including muscle atrophy, osteopenia, hypogonadism, protein-calorie malnutrition, weight loss, and many others [5]. Studies in rats with liver cirrhosis showed that treatments with low doses of IGF-I help to induce significant improvements in intestinal absorption [6], hypogonadism [7], and liver functions [8]. Replacement therapy with IGF-1 in liver cirrhosis patients requires daily doses of 1.5 to 2 mg. Thus, a single patient would need to consume about 600 mg IGF-1per year. However, IGF-1 treatment is very expensive. In addition to the applications described above, IGF-1 is used in treatment of dwarfism [9], diabetes [10] and osteoporosis [11].

Currently, most of the IGF-1 that is available is synthesized in *E*. *coli *[12] or yeast [13]. Construction and maintenance of fermentation systems are very expensive. In addition, formation of inclusion bodies in E. coli or variable biological activities of different forms of IGF-1 in yeast are disadvantages of current production systems. Transgenic plants are good expression systems for large-scale production of recombinant proteins at industrial levels. Plant systems have many advantages including the low cost of growing plants on a large scale, the availability of natural protein storage organs, and the established practices for their efficient harvesting, transporting, storing, and processing [14]. It has been estimated that the cost of producing recombinant proteins in plants could be 10 to 50 fold lower than producing the same protein by *E. coli *via fermentation [15].

However one major drawback of expression of human blood proteins via the nuclear genome is their low levels of expression, mostly less than 1% of the total soluble protein. Some examples of these proteins are human serum albumin 0.02%, haemoglobin 0.05%, and erythropoietin 0.0026% of total soluble protein [16,17]. Also, a synthetic gene coding for the human epidermal growth factor was expressed only up to 0.001% of total soluble protein in transgenic tobacco [17]. IGF-1 expression level in transgenic rice and tobacco was in the range of 22–113 ng/mg protein or 0.002 – 0.011% total soluble proteín, after optimization of codons for plant expression and use of optimal regulatory sequences with or without leader peptides [18]. Although improvements have been made recently for enhancing expression of foreign genes [19], most progress has been made in expression of vaccine antigens [20,21] and monoclonal antibodies [22] using plant viral technology. However, there are not many examples of high level expression of human blood proteins using nuclear transgenic plants. The most commonly encountered challenges are random integration of transgenes into the nuclear genome leading to position effect and transgene silencing, resulting in low levels of foreign gene expression. The position effect could be eliminated by site specific integration of transgenes into the chloroplast genome [23,24]. No gene silencing has been ever reported in transgenic chloroplasts. In spite of expression of transgenes up to 46% of the leaf protein [25] or 150–170 fold higher transcription than the nuclear transgene [26,27], no gene silencing has been observed in transgenic chloroplasts. Yet another major advantage of transgene expression via the chloroplast genome is their containment because of maternal inheritance of chloroplast genome [28,29]. Containment of foreign genes via pollen or seeds is achieved by their expression only in leaves or vegetative tissues and their harvest before emergence of reproductive structures.

Based on these advantages, several vaccine antigens and biopharmaceuticals have been expressed in chloroplasts and their efficacy has been evaluated. For example vaccine antigens have been expressed against bacterial, viral and protozoan pathogens and have been shown to be immunogenic and offer protection against pathogen challenge [30-36]. Similarly, several human blood proteins including somatotropin [37], interferon alpha [38], interferon gamma [39] and insulin [40], were expressed in chloroplasts and shown to be properly folded and fully functional. However, no detailed study has yet been reported on evaluation of the structure of human blood proteins or their amino acid sequence or codon usage. Therefore in this article, we investigate optimization of codon composition of the human IGF-1 and compare expression of the native and synthetic genes at different stages of growth and development of transgenic lines under normal or continuous illumination. After purification of chloroplast derived IGF-1 using IgG sepharose affinity column chromatography, 2-D, electrophoresis and mass spectrometer analyses were used to investigate structural identity. Cell proliferation assay was used to evaluate biological activity of chloroplast derived IGF-1.

## Results

### Chloroplast vectors with native and synthetic IGF-1 genes

**Figure 1:**
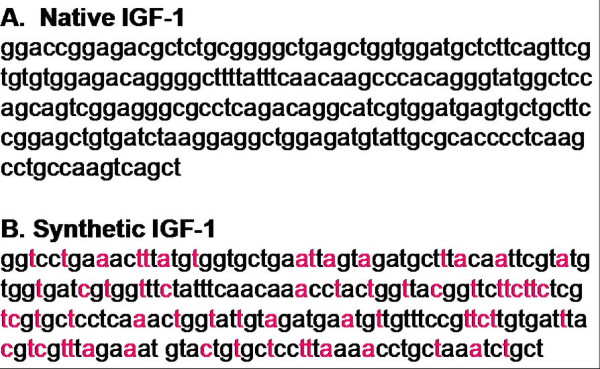
**Nucleotide sequence of *IGF-1 *genes**. A) Nucleotide sequence of the native *IGF-1n *gene. **B**) Nucleotide sequence of the synthetic *IGF-1s *gene optimized for chloroplast expression. Red letters show nucleotides that were modified in the *IGF-1s *gene.

Analysis of the codon composition of IGF-1 gene revealed a less than optimal AT content of 41% for chloroplast expression. The most highly translated protein in the chloroplast is encoded by the *psbA *gene; therefore codon composition of this gene served as a model for IGF-1 optimization (Figure 1). After optimization of the IGF-1 gene, the AT content was increased from 41% to 60%. One goal of this study was to compare expression levels of the native IGF-1 (IGF-1n) gene to the optimized, synthetic IGF-1 (IGF-1s) gene. To test the expression levels of IGF-1n and IGF-1s, tobacco plants were transformed with the chloroplast transformation vector (pLD) containing either the *IGF-1s *or *IGF-1n *gene. The pLD vector contains the homologous recombination sequences *trnI *and *trnA*, that allowed site specific integration into the chloroplast genome as described previously [23,24]. Both the native and synthetic genes contain the *psbA *promoter and 5' UTR, which enhances translation under illumination. The *psbA *5' UTR is a cis acting regulatory element, controlling the translation of genes in higher plants. In addition, both constructs contain a 3' UTR, shown to increase the stability of the transcript [41]. The integration of either IGF-1 gene cassette into the inverted repeat region should double the transgene copy number.

The IGF-1 gene was fused to the ZZ tag to facilitate the purification process. Creating this fusion increases the protein's stability and protects the polypeptide from proteolytic degradation. The pLDG-IGF-1n and pLDG-IGF-1s vectors were designed with the Glu-Asn-Leu-Tyr-Phe-Gln-Gly amino acid sequence, which is recognized by the Tobacco Etch Virus (TEV) protease and cuts between the Gln-Gly. In this way, the IGF-1 polypeptide can be released without any extra amino acids.

### IGF-1 expression in *E. coli *using chloroplast vectors

**Figure 2:**
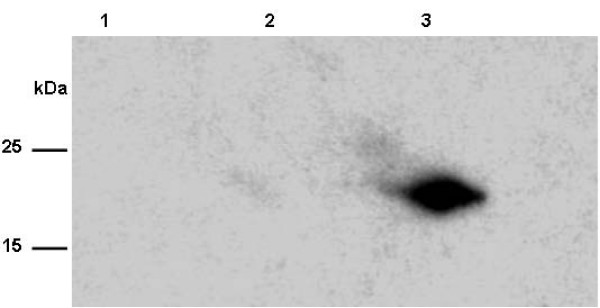
***IGF-1 *Expression in E. *coli***. Expression of IGF-1 in western blots was detected using mouse anti-human IGF-1. Lane: 1-untransformed *E. coli*; Lane 2-pLD5'UTRZZTEVIGF-1n; Lane 3: pLD5'UTRZZTEVIGF-1s. The zz tag-TEV-IGF-1 polypeptide has a molecular size of 24 kDa.

The chloroplast expression system utilizes many prokaryotic features including transcription and translation. Therefore, western blot analysis was used to detect IGF-1 expression in *E. coli*. When the two plasmids, IGF-1s and IGF-1n were tested in *E. coli*, expression of the protein was detected only in clones with the synthetic gene (Figure 2) and not in the native human IGF-1. Therefore this observation confirms that an optimized gene enhances translation in a prokaryotic system (*E. coli*).

### Confirmation of site-specific transgene integration

**Figure 3:**
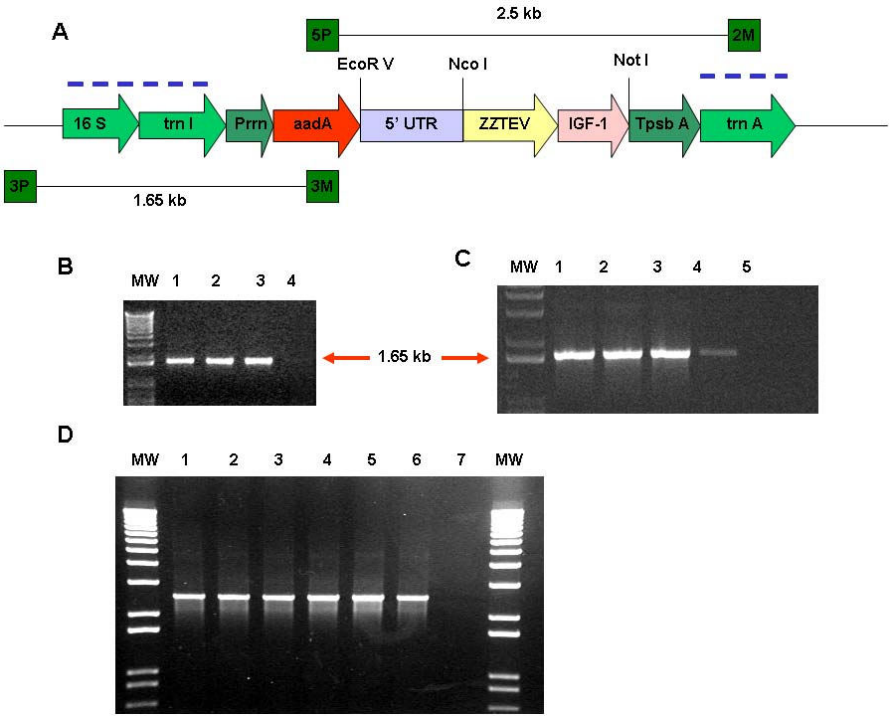
**Chloroplast vectors and PCR confirmation of transgene integration**. A) Two chloroplast expression cassettes were made, one with the *IGF-1n *gene and another with the *IGF-1s *gene (for complete sequence, see figure 1). A) The blue dotted lines show regions of homologous recombination with the chloroplast genome. Regulatory sequences used were from the tobacco chloroplast genome: Prrn: 16S *rRNA *promoter; 5'UTR: the *psbA *promoter and 5' UTR; 3'UTR: the *psbA *3' UTR. The primers 3P & 3M and 5P & 2M were used to confirm the integration of the *IGF-1 *gene cassette into the chloroplast genome. The primer (3P, 3M or 5P, 2M) landing sites for PCR reactions are shown in green boxes. Transgenic lines should produce a 1.65 kb PCR product with 3P & 3M primers and 2.5 kbp products with 5P & 2M primers. B) Lanes 1–3: *IGF-1s *transgenic lines; lane 4: Untransformed control. C) Lanes 1–4: IGF-1n transgenic lines; lane 5: Untransformed control. D) Lanes 1–3: transgenic lines with the 5'UTRZZTEVIGF-1s gene cassette; Lanes 4–6: Transgenic lines with the 5'UTRZZTEVIGF-1n gene cassette. Lane 7: Untransformed control. Lanes marked MW show 1 kbp DNA ladder.

Tobacco leaves were bombarded with the pLDG-IGF-1s and pLDG-IGF-1n vectors. After 48 hours of incubation in the dark, the bombarded leaves were cut into small pieces (5 mm square) and placed in RMOP medium with spectinomycin selection (500 mg/l). This high concentration of spectinomycin helped to eliminate untransformed cells and cells in which the gene cassette integrated into the nuclear genome (because nuclear transformed plants do not produce enough aadA enzyme to overcome such high levels of antibiotic selection). After four weeks of growth on the selection medium, the putative transgenic green shoots appeared from bleached leaves. The 3P and 3M primer pair that land in the native chloroplast genome and in the *aadA *gene, respectively, confirmed integration of the gene cassette into the chloroplast genome (Figure 3A). Transformed plants that have the gene cassette integrated into the chloroplast genome showed a 1.65 kb PCR product (see Figures 3B, C). Plants that grew in the selection medium but did not show transgene integration in the chloroplast genome are most likely mutants, with mutations in the *16S rRNA *gene. Transgenic shoots that show the 1.65 kb PCR product have the gene cassette integrated into the chloroplast genome. Those shoots that produced the correct size PCR product were cut into small pieces and transferred into fresh RMOP medium with spectinomycin for a second round of selection. After transgenic shoots were obtained from the second round of selection, they were tested with the 5P-2M primers to confirm the integration of the *aadA *and *IGF-1 *genes into the tobacco chloroplast genome. The positive transgenic shoots produced a 2.5 kb PCR product (see Figure 3D).

### Evaluation of homoplasmy

**Figure 4:**
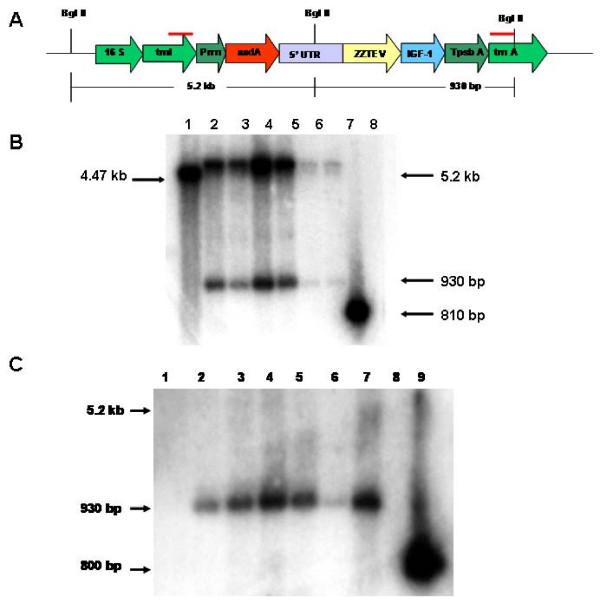
**Southern blot analysis**. A flanking sequence probe (810 bp) was obtained from the *trnA*/*trnI *genes (indicated by red lines in 4A). Regulatory and coding sequences are the same as in figure 3A. This map also shows the chloroplast DNA fragment that hybridizes with this probe. Untransformed genome hybridized with a 4.47 kbp fragment whereas transformed chloroplast genomes hybridized with two fragments of 5.2 kbp and 930 bp. B) Lane 1: Untransformed control; Lanes 2–3: *IGF-1s *transgenic lines (T_0 _generation); Lanes 4–5: *IGF-1s *transgenic lines (T_1 _generation); Lanes 6–7: *IGF-1n *plants (T_0 _generation); Lane 8: positive control. C) The *IGF-1 *coding sequence was used as a probe to confirm integration of the *IGF-1 *gene into the chloroplast genome. The transgenic lines that contain the *IGF-1 *show a 930 bp fragment. Lane: 1- untransformed, Lanes 2–3: *IGF-1s *transgenic lines (T0), 4–5: *IGF-1s *transgenic lines (T1); 6–7: *IGF-1n *transgenic lines; Lane 8: blank; Lane 9: the IGF-1 probe as a positive control.

The potted plants were tested by Southern blot analysis to evaluate if transgenic lines were homoplasmic or heteroplasmic. The flanking sequence probes allowed us to identify if all the chloroplast genomes are transformed (homoplasmic) or if both the transformed and untransformed chloroplast genomes were present (heteroplasmic). This probe contained portion of the *trnI *and the *trnA *genes and therefore, the probe hybridized with the *trnI *and *trnA *genes that are in the chloroplast genome (Figure 4A). The transgenic and untransformed plant DNA were digested with *Bgl II *restriction endonuclease, which produced two DNA fragments (5.2 kb and 0.93 kb) in transgenic plants and one fragment of 4.47 kb in untransformed plants. The T_0 _transgenic plants containing the IGF-1s and the IGF-1n showed only the two fragments of the transgenic chloroplast (5.2 kb and 0.93 kb), confirming that these plants had achieved homoplasmy (Figure 4B). The T_1 _IGF-1s plants were also homoplasmic [seeds were germinated on MSO with very high concentration of (500 mg/l) of spectinomycin]. A second probe (IGF-1 probe) was used to confirm the integration of the *IGF-1 *gene into the chloroplast genome of these transgenic lines (Figure 4C). All of the transgenic lines showed the 930 bp fragment produced by the *Bgl II *digestion confirming that the *IGF-1 *gene was integrated into the chloroplast genome. Untransformed plants did not show this fragment in the Southern blots.

### Evaluation of chloroplast transcripts

**Figure 5:**
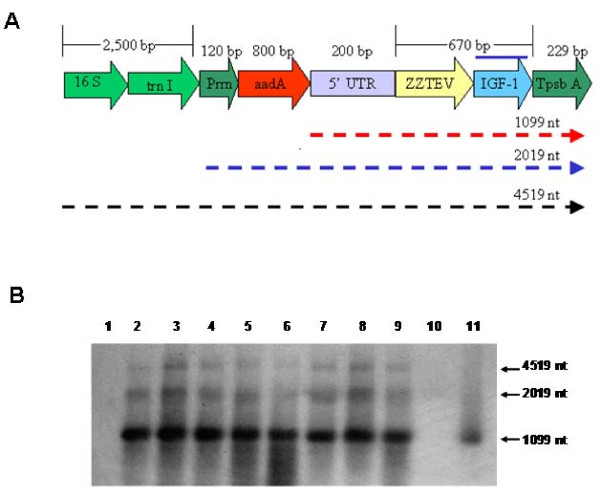
**Northern blot analysis**. A) The map of pLD-5'UTR-ZZTEVIGF-1 shows a monocistron transcript of 1099 nt, a dicistron transcript of 2019 nt, and polyciston transcript of 4519 nt. B) Lane 1: untransformed control. Lanes 2–3: 5'UTRZZTEVIGF-1s transgenic lines (T0); Lanes 4–7: 5'UTRZZTEVIGF-1s transgenic lines (T1); Lanes 8–9 5'UTRZZTEVIGF-1n Protein quantification by ELISA in (T0); Lane 10: blank; Lane 11: *IGF-1 *probe used as a positive control.

The potted plants were grown in a photoperiod of 16 hours of light and 8 hours of dark at 27°C. RNA was extracted from transformed and untransformed tobacco plants to perform northern blot analysis. Transcripts of about 1099 nucleotides were observed in the transgenic lines, which contains the *psbA *promoter, 5' UTR, the *IGF-1 *gene, and *psbA *3' UTR. This mRNA is considered monocistronic and it is the most abundant transcript in all transgenic lines (Figure 5A, B). In addition, dicistronic and polycistronic transcripts were observed in lower abundance in the chloroplast transgenic lines. Northern blot analysis also showed that the IGF-1s and the IGF-1n transgenic lines had similar transcript abundance and there were no significant differences at the transcriptional level between the *IGF-1n *and *IGF-1s *genes. Also, unusual transcripts were not observed in the native gene, confirming the lack of non-specific processing of transcripts.

### IGF-1 Expression in Transgenic Chloroplasts

**Figure 6:**
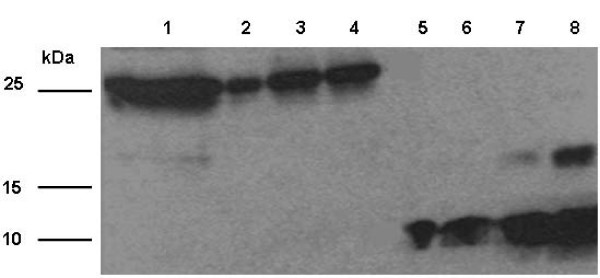
**Western Blot Analysis**. The plant samples were run in 12% SDS-PAGE and the blot was detected using mouse anti-human IGF-1. Lane 1: 5'UTR-ZZTEV-IGF-1n transgenic line. Lane 3: T_0 _5'UTR-ZZTEV-IGF-1s transgenic lines. Lane 4: T1 5'UTR-ZZTEV-IGF-1s transgenic lines. Lanes 5–8: IGF-1 standards with a concentration of 10 ng, 25 ng, and 50 ng.

Western blots made using protein extracts from plants grown in a photoperiod of 16 hours of light and 8 hours of dark showed that the plants transformed with *IGF-1s *and *IGF-1n *genes were expressing the IGF-1 polypeptide, which had a molecular weight of 24 kDa (Figure 6). Autoradiographs were used to quantify the amount of IGF-1 expressed in the different transgenic lines using Alpha Imager and AlphaEase FC software (Alpha Innotech) by comparison with known quantities of IGF-1 standard. The IGF-1n plants had an expression level of 10.9% IGF-1 in the total soluble protein (%TSP). The IGF-1s plants (T_0_) had a 12.5% TSP and the T1 plant (T1 plant is a younger plant and T_0 _is a mature plant) had a 4.8% TSP. Enzyme-linked immunosorbent assays (ELISA) were performed in the same transgenic lines to further confirm protein quantification. ELISAs showed that IGF-1n transgenic lines had an expression level of 9.5% TSP. The IGF-1s transgenic lines (T_0_) had 11.3% TSP and the T1 transgenic lines had 4.9% TSP (Figure 6, 7A). Thus, expression levels were confirmed by both methods.

**Figure 7:**
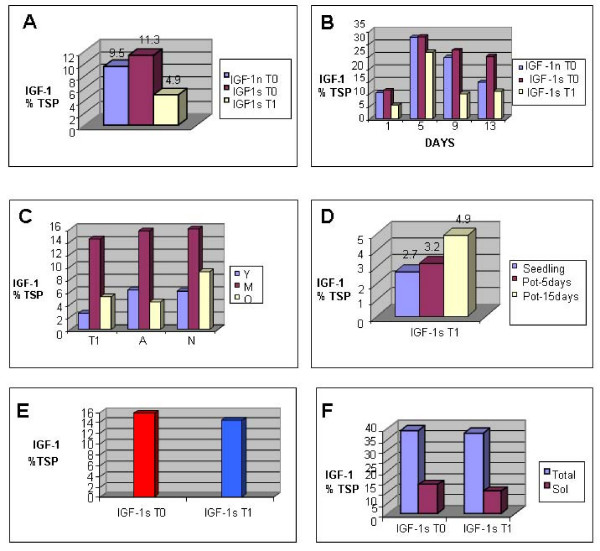
**IGF-1 expression in transgenic chloroplasts**. ELISAs show IGF-1 expression as percentage of the total soluble protein. A) Transgenic lines grown in a 16 hours light and 8 hours dark photoperiod; T_0 _are mature plants and T_1 _is a younger plant. B) Transgenic lines grown in continuous light for 13 days; C) Protein quantification by ELISA in young (Y), mature (M), and old (O) transgenic leaves. Young leaves were among the top few leaves; mature leaves were fully grown, present in the middle of the plant; the bottom few scenescing leaves were identified as old. D) Protein quantification by ELISA in seedlings and potted plants grown for 5 days and 15 days. E) IGF-1 expression in *IGF-1s *T_0 _and T_1 _transgenic lines. F) IGF-1s present in the total and soluble fractions of T_1 _and T_0 _generations.

The transgenic tobacco lines were exposed to continuous light for 13 days to evaluate IGF-1 expression levels, because the *psbA *promoter and 5' UTR are regulated by light. ELISAs showed more than 2 fold increase in the expression levels after the transgenic lines were exposed for 5 days of continuous light (Figure 7B). The IGF-1s transgenic line (T_0_) had an IGF-1 expression level of 32.7% TSP and T_1 _transgenic line had 26.6% TSP, because these were younger plants. The IGF-1n-plant (T_0_) had an expression level of 32.4% TSP. The expression levels were measured again after 9 and 13 days. For both IGF-1s and IGF-1n, the ELISAs showed a decrease in the expression levels (Figure 7B), although the decrease was more significant in IGF-1n transgenic lines. Additionally, IGF-1 protein accumulation was measured in young, mature, and senescing leaves. A young leaf was taken from the top five leaves, the mature leaf was green and fully-grown from the mid-section of the plant, and the old leaf was senescent and from the very bottom of the plant. Figure 7C shows that all transgenic lines had a higher IGF-1 expression in mature leaves. Younger leaf cells contained fewer chloroplasts and the *psbA *was developmentally regulated; therefore, expression levels were less than mature leaves. Older senescent leaves had lower accumulation of IGF-1 probably due to higher proteolytic activity. Another experiment was performed to quantify IGF-1 expression during plant development by comparing seedlings and plants after 5 days and 15 days of growth in pots. The transgenic tobacco line showed an IGF-1 expression level of 2.71% TSP in the seedling, 3.2% TSP after 5 days in the pot and 4.9% TSP after 15 days in the pot (see Figure 7D), confirming developmental regulation of the *psbA *promoter. ELISA experiments showed that the expression levels between the T_0 _and the T_1 _transgenic lines were very similar (Figure 7E). Quantitation of IGF-1 in different fraction showed that it was present in both the total and soluble fractions (Figure 7F), suggesting that some of the IGF-1 is in the insoluble fraction.

### Purification and characterization of IGF-1 expressed in chloroplasts

**Figure 8:**
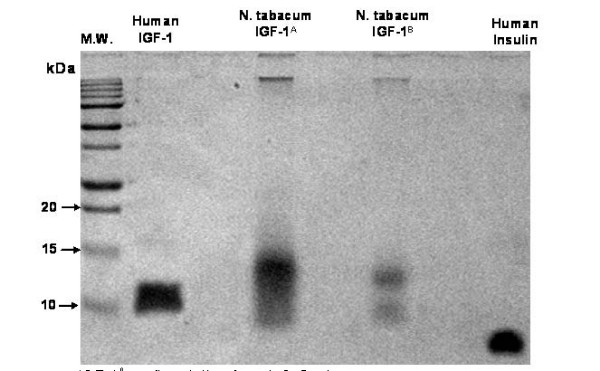
**Chloroplast derived IGF-1 before and following hydroxylamine treatment**. Transgenic tobacco IGF-1 was purified from centrifuged plant homogenates by IgG-sepharose affinity column chromatography. Proteins eluted from the IgG-sepharose column were identified by polyacrylamide gel electrophoresis. Lanes from left to right: molecular weight marker proteins (MW), human insulin like growth factor 1 (human IGF-1), tobacco chloroplast-derived human IGF-1, (IGF-1A). Darker upper broad band of approx. 15 kDa = IGF-1 linked to *Staphylococcus aureus *Z domains. Partial removal of *S. aureus *Z domains by hydroxylamine cleavage (IGF-1B), lower broad band = IGF-1. Right lane = human insulin.

Human insulin like growth factor (IGF-1) linked to the *S. aureus *Z tag was isolated by IgG Sepharose affinity column chromatography and examined by polyacrylamide gel electrophoresis for purity (Figure 8). Partial removal of the *Staphylococcus aureus *Z domains from chloroplast-derived IGF-1 was obtained following hydroxylamine cleavage of the S. aureus Z domain – IGF-1 fusion protein (Figure 8). Immunoblot analysis using human anti-IGF-1 as the primary antibody identified the two bands as containing IGF-1 (Figure 8).

**Figure 9:**
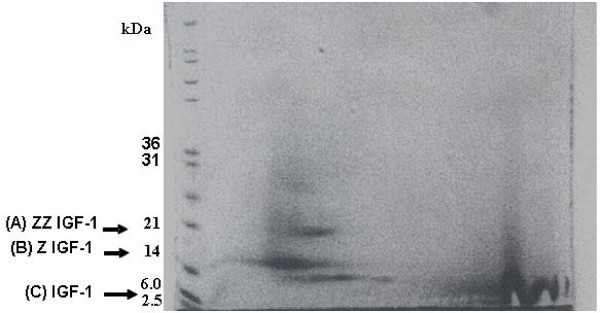
**The IGF-1 proteins isolated from the IgG-sepharose column were separated on a 2-D focusing/phoresis acrylamide gel**. Five μg of the IGF-1 – *S. aureus *fusion protein was loaded on a 10% bis-acrylamide gel for electrofocusing and phoresis. Electrofocusing was conducted initially over a pH range of 4–10. Following isoelectric focusing and electrophoresis, four proteins (6, 7, 14 and 25 kDa) were detected after Coomassie Blue staining. These proteins corresponded in molecular weight to ZZ-IGF-1 (A), Z-IGF-1 (B) and IGF-1 (C).

**Figure 10:**
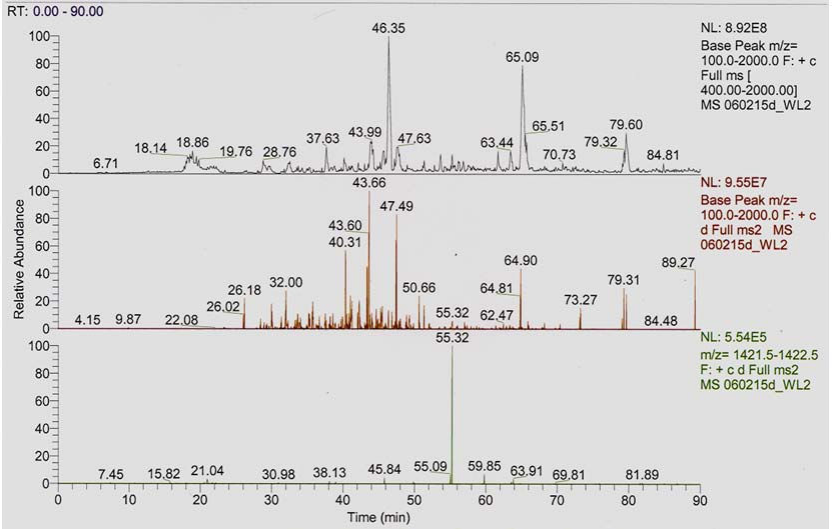
**Mass Spectrometry Analysis of Chloroplast Synthesized Human IGF-1**. The 7, 14 and 25 kDa protein bands were trypsin-digested and evaluated by mass spectrometry. The top panel is the pure MS without fragmentation. The second panel shows all of the significant MS/MS events derived from the MS scans. The third panel shows the MS/MS events that yielded a fragment with m/z 1421 +/- 1 mu. These were all grouped together in one cluster eluting at 55.32. S. aureus protein A was found in all spots examined and the active portion of IGF-1 was identified. In at least two of the spots analyzed, human IGF-1 and *S. aureus *Z-tag was detected. Shown are full MS and MS/MS patterns for discovery of peptide 105–116 with an elution time of 55.32 for a single charge mass of 1421.1 and a double charge mass of 711.6.

To confirm IGF-1 structure by mass spectrometry, 2D focusing/phoresis was performed on the chloroplast synthesized IGF-1 protein. Four protein spots were detected at 6, 7, 14 and 25 kDa after staining with Commassie Blue (Figure 9). The 7, 14, and 25 kDa spots were digested with trypsin and evaluated for the presence of IGF-1 peptides by MS/MS mass spectrometry using a Thermo LCQ Deca XP mass spectrometer fitted with a nanospray RP column (Figure 10, all panels). Evaluation of the spectra was accomplished with X-Tandem software matching against the fasta databases for *S. aureus *and *H. sapiens*. The fasta databases provide in simple single letter format, the primary protein sequences contained in the explored sample . In figure 10, the top panel is the pure MS without fragmentation. The second panel shows all of the significant MS/MS events derived from the MS scans. The third panel shows the MS/MS events that yielded a fragment with m/z 1421 +/- 1 mu. These were all grouped together in one cluster eluting at 55.32. The elution time of 55.32 shows a single charge mass of 1421.1 and a double charge mass of 711. 6. The X-Tandem software identified *S. aureus *protein A in all the spots examined (Figure 10, top and middle panels). Sequest software (University of Washington, Seattle) permitted identification of the active portion of IGF-1B and identified 5 tryptic peptides yielding dozen ions ranging from M/Z 326 to 2306 (Figure 10, top and middle panels). The heaviest spot on the 2D gel (25 kDa) gave an IGF-1 signal on three peptides from this spot. Of particular significance were signals generated by peptide 70–84 (GFYFNKPTGYGSSSR yielding 1669, 834 and 557 ions) and peptide 105–116 (LEMYVAPLKPAK yielding 1422 and 711.6 ions (Figure 10). Signals for these peptides were also found in the second heaviest 14 kDa band (Figure 10). Thus, in at least two of the spots analyzed from the 2D gel separation of the IgG sepharose column eluate, it was possible to identify both human IGF-1 and the *S. aureus *Z tag domain (Figure 10).

**Figure 11:**
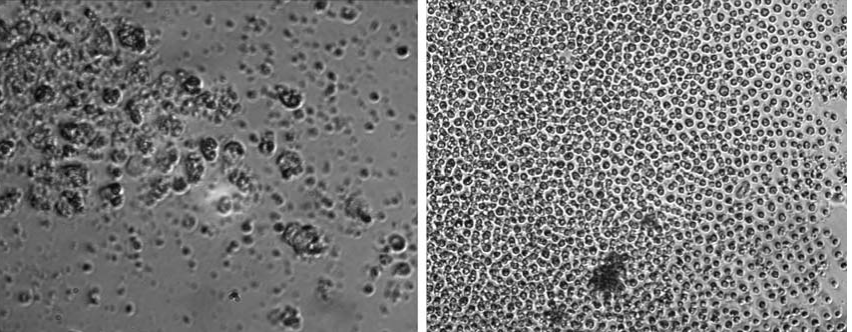
**Chloroplast synthesized human IGF-1 stimulates the growth and proliferation of human cells in culture**. Proliferation of human megakaryoblastic HU-3 cells were investigated by phase contrast light microscopy at 4 and 7 days after the addition of huIGF-1. Left panel: Four day HU-3 cell culture to which no chloroplast synthesized IGF-1 was added (400 × magnification). Right panel: HU-3 cell culture of the same age to which 62 ng/ml chloroplast-derived, partially purified IGF-1 was added at time zero (100 × magnification).

**Figure 12:**
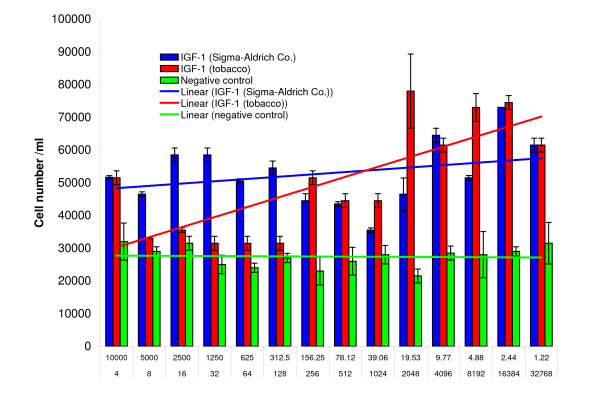
**Effect of chloroplast-derived IGF-1 on HU-3 cell proliferation**. IGF-1 was added to HU-3 cells; 48 hours later, cell proliferation was measured by the Cy-QUANT NF Cell proliferation assay (Invitrogen) and by hemocytometer counting. Proliferation was measured by fluorescence determination of DNA replication based on intercalation of a fluorescent dye into double stranded DNA. Cell proliferation was determined according to the instructions supplied by the manufacturer. Red (chloroplast derived IGF-1), blue (Sigma IGF-1), and green (negative control) lines represent the best fit of the respective data points for each proliferation assay treatment. Top row shows concentration (ng/ml) of IGF-1 (Sigma). Bottom numbers are dilution factor of chloroplast-derived IGF-1. Negative control is the dilution of the TS buffer.

The biological activity of partially purified human IGF-1 preparations isolated from tobacco chloroplasts was established by measurement of IGF-1 stimulation of mammalian cell proliferation. Briefly, human megakaryoblastic (HU-3) cells carrying the IGF-1 receptor were cultured in RPMI-1640 medium. At the beginning of the time course assay, commercial human IGF-1 (Sigma), or IGF-1 partially purified from tobacco chloroplasts was added to each well at several different concentrations. The cell cultures were incubated at 37°C for 48 hours and a portion of each culture was stained with Trypan Blue dye to quantify cell viability prior to cell proliferation measurements as reliably determined by hemocytometer cell counting methods. Murine 3T3 fibroblasts carrying the IGF-1 receptor were maintained in DMEM medium and were evaluated in different concentrations of IGF-1 by the CyQUANT NF Cell proliferation assay (Invitrogen Inc., San Diego, CA) and by hemocytometer counting methods. Cell proliferation was measured by fluorescence measurements of DNA replication based on intercalation of a fluorescent dye™ into double stranded DNA. While increased numbers of mouse 3T3 cells were detected as compared to the uninoculated control, this increase was significantly less than the increase in human HU-3 cell numbers stimulated by plant synthesized IGF-1 (Figure 11). Red, blue and green lines represent the best fit of the respective data points for each proliferation assay (Figure 12). At the beginning of the time course assay, commercial human IGF-1 with purity >97 (Sigma I3769) or partially purified chloroplast-derived IGF-1 or TS buffer (as a negative control) was added to cells in 3 wells at different concentrations. The error bars represent growth differences among these wells of the tissue culture plate. Chloroplast-derived IGF-1 was tested starting from dilution 1:4, which corresponded to 10,000 ng/ml IGF-1. Addition of chloroplast-derived IGF-1 resulted in a dose-dependent growth response at low concentrations. The effective range was in dilutions at 1: 2000 – 1: 32 000, which corresponded to 20 -1 ng/ml IGF-1. Worthy of note, is the observation that the maximal level of the cell proliferation was seen with commercial IGF-1 (Sigma) almost at similar concentrations.

Chloroplast-derived IGF-1, at higher concentrations (dilutions between 8 –128) did not significantly stimulate the proliferation of the HU-3 cells; this could be due to some impurity present in these preparations that were obtained with rapid purification. However, >97% pure commercial IGF-1 also did not show dose dependent increase between 300 ng/ml and 10,000 ng/ml concentrations. These results suggest that both the commercial and plant synthesized IGF-1 have similar levels of mitogenic activity and that the apparent dip in the dose response curve at higher IGF-1concentrations may be due to currently unknown environmental factors that had an approximately equal effect on the two sources of IGF-1. Therefore, addition of chloroplast-derived IGF-1 resulted in a dose-dependent growth response of HU-3 cells, very similar to commercial IGF-1. Alterations in the mitogenic response to decreasing IGF-1 dosage are similar in both chloroplast-derived IGF and commercial IGF inoculated cell cultures. These observations argue against specific differences between chloroplast-derived and commercial IGF-1. Chloroplast-derived IGF-1 functioned efficiently in spite of the presence of ZZ-tag and possibly other impurities present after rapid purification.

## Discussion

Transplastomic lines express significant amounts of human IGF-1. The difference in IGF-1 expression levels is insignificant between the synthetic and native genes in the chloroplasts. This may be due to optimal levels of expression already achieved with the native gene and limitations of the chloroplast protein synthetic machinery other than codon usage. On the contrary, the IGF-1 polypeptide was only expressed in the *E.coli *cells that contain the *IGF-1s*. These results show that *E. coli *translational machinery may be different from chloroplast in codon preference and usage. Although previous studies used chloroplast vectors for expression of foreign proteins in E. coli, this is the first time a dramatic difference has been observed between these two systems in their translation machinery.

The *psbA *promoter and 5'UTR were used in the pLDG-IGF-1n and pLDG-IGF-1s vectors to enhance the protein expression. The expression levels increased more than 2 fold after five days in continuous light. Also, IGF-1 expression levels increased during plant development. Both light regulation and developmental regulation of the *psbA *gene is well known in the literature although this study has further confirmed the role of such regulatory elements using a human blood protein. This information will be useful in various biotechnology applications. Similarly, IGF-1 expression was measured in young, mature and old leaves. The IGF-1 expression was higher in mature leaves and this again provides an ideal time to harvest this protein in the green house or field.

Maternal inheritance of genetically modified chloroplast genomes and the absence of any reproductive structures when foreign proteins are expressed in leaves, offer efficient transgene containment and facilitates their safe production in the field [28,29]. Two recent studies point out efficient control of maternal inheritance of transgenes in transplastomic tobacco. Ruf et al [42] set up a stringent selection system for paternal transmission by using male sterile maternal parents and transplastomic pollen donors conferring plastid specific antibiotic resistance and green fluorescence for visual screening. This selection system identified six among 2.1 million seedlings screened (frequency of 2.86 × 10-^6^) that showed paternal transmission of transgenes and the authors concluded that plastid transformation provides an effective tool to increase biosafety of GM crops. Svab and Maliga [43] examined the contribution of alien cytoplasm to rare paternal plastid transmission and reached similar conclusions. Transplastomic plants producing human therapeutic proteins have been already tested in the field after obtaining USDA-APHIS approval [38]. Because of such unique advantages, transgenic chloroplasts have been used for expression of human therapeutic proteins [30-40,44].

IgG-Sepharose affinity column chromatographic separation of Z domain containing chloroplast derived IGF-1 protein confirmed a protein with a molecular mass corresponding to human IGF-1. Staphylococcal protein A (SPA) is an immunoglobulin-binding receptor present on the surface of the gram positive bacterium Staphylococcus aureus. The strong and specific interaction between SPA and the constant part (Fc) of certain immunoglobulins (Ig) have made it useful for purification and detection of immunoglobulins in a variety of different applications [45,46] and therefore in our study the ZZ tag was used for purification of IGF-1 fusion proteíns expressed in chloroplasts. SPA does not contain any cysteine residues that could interfere with the disulfide formation within a fused target protein [47]. The hydroxylamine cleavage resulted in approximately 40% cleavage of the ZZ tagged IGF-1 to IGF-1 + ZZtag-IGF-1 as observed by acrylamide gel staining following gel electrophoresis. Hydroxylamine cleavage was more efficient than TEV protease and less expensive but resulted in full length IGF-1 with no extra amino acids. There is evidence that dependent upon the fusion protein, the ZZ tag can be more or less resistant to hydroxylamine cleavage. This may be responsible for the reduced level of ZZ-tag cleavage [48].

## Conclusion

The presence of human IGF-1 in purified plant extracts was confirmed by single and two dimensional electrophoresis methods and the structural elements of chloroplast-derived IGF-1 was further confirmed by mass spectrometer analysis. Cell proliferation assays confirmed the biological activity of chloroplast derived IGF-1 and found that human HU-3 cells react strongly to the addition of chloroplast generated IGF-1 even in the presence of the *S. aureus *Z tag. Proper functionality of IGF-1 suggests that required disulfide bonds are formed. Presence of disulfide bonds in several chloroplast-derived human blood proteins including interferon alpha, gamma and somatotropin [37-39] was evaluated by their functionality in cell culture assays. Therefore, therapeutic proteins expressed in transgenic chloroplasts have proper post-translational modifications and are fully functional. While purification of chloroplast generated human IGF-1 to homogeneity remains to be attained, retention of significant biological activity of the ZZ tagged IGF-1 molecule indicates its effective biological usefulness even in an altered state. Therefore, the ability to use plant chloroplasts as bioreactors to generate proteins of great economic value that retain their essential biological activity is an exciting goal that appears to be within our grasp.

## Methods

### Recursive PCR and Primer Design

For synthesis of optimized IGF-1 (IGF-1s) gene, four primers were designed: two external primers of 56 bp and two internal primers of 100 bp. All the primers have an overlapping region of 20 bp. The 5' external primer was engineered to include the sequence of the TEV enzymatic cleave site and the 3' primer contained the *NotI *restriction site. In recursive PCR reaction, the external oligonucleotides were in higher concentration than the internal (20–30 pmol of the external primers and 0.2–0.3 pmol of the internal primers). The lower concentration of the internals oligonucleotides assisted in avoiding unwanted products.

Two different parts were used in the recursive PCR [49,50]. In the first part, the reaction were run through 10 cycles using the following temperature sequence: 94°C for 30 seconds to denature the DNA, 55°C for 30 seconds for annealing primes, and 72°C for 1 minute to synthesize DNA. An incubation period of 7 minutes at 72°C followed after the cycles ended. The primers were designed to have an annealing temperature of 55°C to avoid unspecific binding of the primers. The second part consisted of 30 cycles, denaturing the DNA for 30 seconds at 94°C, then primers annealing for 30 seconds at 65°C, followed by DNA synthesis for 7 minutes at 72°C. The PCR product was run on 1.5% agarose gel at 65 volts for 55 minutes to visualize amplified products. The IGF-1s was cloned into the pBluescript KS II, and E. *coli *cells were transformed with this vector.

### Bombardment and Selection of Transgenic Lines

Sterile leaves were bombarded using the Bio-Rad PDS-1000/He biolistic device [51,52]. The bombarded leaves were incubated in the dark for 48 hours and then cut and placed in RMOP medium with 500 μg/ml of spectinomycin.

### PCR Analysis

The plant DNA was extracted from leaves using the Qiagen Dneasy Plant Mini Kit (Quiagen). The 3P and 3M primers were used to perform PCR on transformed and untransformed plants [51-53]. Samples were run for 30 cycle with the following sequence: 94°C for 1 min., 65°C for 1.5 min., and 72°C for 2 min. PCR products were analyzed on 0.8% agarose gel.

### Southern Blot Analysis

The plant DNA of the transgenic and wild type tobacco plants were digested with *Bgl*II, and separated on 0.8% agarose gel and transferred to a nylon membrane. The 0.8 kb probe was generated by digesting pLD-CtV2 (that contains the trnI and *trnA *genes) vector with *BamH*I *and Bgl*II and was labeled with ^32^P (Amersham). The probe was hybridized with the membrane using the QUICK-HYB hybridization solution and protocol (Stratagene).

### ELISA

ELISA was used to quantify the IGF-1 expression levels in different transgenic lines. Different concentrations of 100 mg leaves (transformed and untransformed plants) were ground with liquid nitrogen. Bicarbonate buffer, 500 μl, pH 9.6 (15 mM Na_2_CO_3_, 35 mM NaHCO_3_, and 0.1% Tween 20, pH 9.6) was used to resuspend the ground mixture and incubated overnight at 4°C. Diluted sample (1:3000) was added in each well (100 μl) of the plate and this was done in duplicate. Bicarbonate buffer was used as blank. The plate was incubated overnight at 4°C. After washing the wells thrice with washing buffer, PBST (PBS and 0.05% Tween 20), mouse anti human IGF-1 diluted 1 μg/ml in 0.01 M PBST containing 0.3% milk (100 μl/well) was added and incubated for 2 h at 37°C. The wells were washed and incubated with 1:10,000 goat anti mouse IgG-alkaline phosphatase conjugate in 0.01 M PBST containing 0.3% milk (100 μl/well) for 2 h at 37°C. The plate was developed with TMB substrate (100 μl/well, American Qualex) for 30 minutes at room temperature and the reaction was stopped by addition of 50 μl/well of 2 M sulfuric acid and the plates were read at 405 nm. For a standard curve, purified commercially available human IGF-1 (R&D Systems) was diluted with bicarbonate buffer to concentrations between 3 and 25 ng/ml and processed as above. Total soluble plant protein concentration was determined using the DC Protein Microassay (Bio-Rad). IGF-1 expression levels were calculated as a percentage of the total soluble protein.

### Immunoblots

One hundred mg of tobacco leaves were ground in liquid nitrogen and resuspended in 200 μl of extraction buffer (200 mM Tris-HCl, pH 8.0, 100 mM NaCl, 10 mM EDTA, 4 mM PMSF) [54]. Leaf extracts were boiled for 5 minutes in the sample buffer (0.5 M Tris-HCl, pH 6.8, 2.5 ml glycerol, 10% SDS, 0.5% bromophenol blue reached a total volume 9.5 ml with water) (Bio-Rad). All samples were electrophoresed in 15% resolving and 4% stacking gels using the buffer system of Laemmli. The membrane was blocked for 20 minutes at room temperature with PBS and 3% non-fat milk (PBS-milk). Then, the blot was incubated with anti-IGF-1 (Upstate Biotechnology, diluted in PBS-milk until it achieved a final concentration 1 μg per ml) overnight at 4°C. The membrane was washed twice with water. The secondary antibody used was a Goat Anti Mouse IgG conjugated to Horseradish Peroxidase (American Qualex Antibodies) at a 1:5000 dilution, and was added to membranes in blocking solution and incubated for one hour. The blot was washed with water. A final wash was done for 5 minutes in PBS with 0.05% Tween 20. Development was performed by the chemiluminescent method (Pierce).

Following elution from the immuno-affinity column, the protein samples were concentrated by speed vac evaporation and diluted 1:2 in 1× Gel-loading buffer (50 mM Tris, 2% SDS, 0.05% Brome-Phenol Blue, 10 v/v% glycerol and 2 μl DTT (dithiothreitol). The samples were heated at 95°C for 5 min, and 20 μl (protein conc. 500 μg/ml) of the sample was loaded onto the gel. Electrophoresis was performed at 160 V-, in 1× Laemmli running buffer (5× Laemmli: 500 ml-in water solution 7.5 g Tris, 47 g glycine, 2.5 g SDS). Capillary blotting of proteins was carried out overnight in transfer buffer (3 g Tris, 14.4 g glycine, 200 ml methanol per liter). The nitrocellulose membrane was washed for 5–10 min with TST-buffer (4.5 g NaCl, 250 μl Tween 20, 25 ml in 500 ml 1 M Tris, pH 7.5), then blocked in TST containing 5% dry milk (TST = 5 g dry milk in 100 ml) for 30 min in glass tubes of the DNA hybridization equipment, with slow rotation. The primary antibody (goat a-human IGF-I, SIGMA Cat#8773) was diluted1:1250 in TST (with 2.5% dry milk); membranes were rinsed in distilled water. The primary antibody (10 ml) was added to membranes, and incubated for 2 h at room temp with gentle shaking. Membranes were washed 5 times in distilled water followed by 3 × 5 times in TST, (50 ml). The secondary antibody (Rabbit a-goat alkaline phosphatase-conjugate, Sigma Cat#**4049)**, or monoclonal a-goat alkaline phosphatase-conjugate- Sigma Cat#**8062**) was diluted in TST buffer (1:10000, 20000, 50000), added to blots and incubated with gentle rotation on an orbital shaker for 1 h. Blots were washed 3 × 5 times in TST, followed by 5 times in distilled water. Immunoblots were developed for 20–40 min in NBT substrate: made up of 5 mg NBT (nitrotetrazolium blue, Sigma) diluted in100 μl of 70% DMF (dimethyl-formamide) solution; 82 μl BCIP (5-bromo-4-chloro-3-indolyl phosphate disodium salt) was added to 10 ml substrate buffer (100 ml: 10 ml 1 M Tris, 2 ml 5 M NaCl and 5 ml 1 M MgCl_2_). Blots were stained at 4°C overnight. The reaction was stopped by dilution of the substrate in cold tap water.

### Purification of IGF-1 from transplastomic leaves

Transformed tobacco leaves (50 g) were ground to a fine powder in a liquid nitrogen in pre-cooled mortar. The plant powder was thawed by addition of two volumes (100 ml) of cold Extraction Buffer A (50 mM Tris HCl, pH 8.0, containing 200 mM NaCl, 100 mM EDTA and 1 mM phenylmethylsulfonyl fluoride (PMSF) and 0.1% Nonidet P-40). The homogenate was centrifuged in 50 ml Oakridge tubes at 9,000 rpm, 10 min, at 4°C in a pre-cooled SA-600 rotor, in a Sorvall RC-5B centrifuge to remove cell debris. The non-IGF-1 plant proteins in the supernatant were precipitated by the addition of solid ammonium sulfate to 30% saturation (w/v), while the IGF-1 remained in the soluble fraction. The mixture was centrifuged at 9,500 rpm in a Sorvall SA-600 rotor at 4°C for 30 min. The supernatant containing the IGF-1 was raised to 65% saturation with ammonium sulfate and the mixture was centrifuged as before. The IGF-1 was now precipitated in the pellet. The pellet was resuspended in a small volume (3–4 ml) of Extraction Buffer B (25 mM Tris-HCl, pH 7.5, 0.01% thiodiglycol and 10 μM PMSF) – 10×. The mixture was dialysed against 1.0 liter of extraction buffer B at 4°C for 6 hrs in dialysis tubing with a molecular weight cutoff of 7,000 kDa or less. The dialysate was centrifuged as before at 9,500 rpm in the SA-600 rotor for 15 min to sediment insoluble proteins. The supernatant containing the IGF-1 was adjusted to 0.15 M NaCl and loaded onto a IgG-Fast Flow 6 Sepharose (Sigma) immunoadsorbent column (10 ml bed vol), equilibrated with TST buffer (50 mM Tris-HCl, pH 7.6, 120 mM NaCl, 0.05% Tween 20). Proteins other than ZZ-IGF were eluted from the column by washing with 10–20 column volumes of TST buffer. The Tween 20 detergent was removed from the column by washing with 2 volumes of 5 mM ammonium acetate, pH 4.8. The ZZ-IGF-1 fusion protein was eluted from the column with 0.4 M acetic acid, pH 3.4 at a low linear flow rate (20 cm/hr) to obtain a sharp IGF-1 peak. The column eluate was collected (2.0 ml fractions), and monitored (OD_280_) for the appearance of ZZ-IGF-1 protein which eluted after the column volume. The fractions containing the ZZ-IGF-1 protein were pooled and the protein concentration was determined spectrophotometrically by comparison of the OD_260/280 _nm ratio, or more precisely by Bradford protein assay. Residual salt was removed by dialysis for 2–3 hrs against 1 l of 5 mM ammonium acetate, pH 6.0, at 4°C using dialysis tubing with a 3.5 kDa cutoff (Pierce). A sample of the ZZ-IGF-1 protein was subjected to electrophoresis on a 15% acrylamide gel to examine the molecular weight of the ZZ tagged IGF-1.

The ZZ-linker was cleaved from the IGF-1, by dissolving 2.0 mg of previously isolated tobacco ZZ-tagged IGF-1 fusion protein in 1.0 ml of ZZ Buffer without hydroxylamine (0.2 M Tris-HCl, pH 9.2, 1 mM EDTA). Cleavage of the ZZ moiety from the IGF-1 was carried out by addition of an equal volume of ZZ buffer (0.2 M Tris-HCl, pH 9.2, 2 M hydroxylamine, 1 mM EDTA).containing 2.0 M hydroxylamine. The mixture was incubated at 45°C for six hours and the reaction was terminated by lowering the pH to 6.0 with acetic acid. Cleaved IGF-1 was estimated by separation of the two forms by acrylamide gel electrophoresis. Hydroxylamine was removed from the mixture by first lowering the pH to 6.0 with glacial acetic acid (measured with pH paper), followed by dialysis of the mixture for 2 hrs at 4°C in 1.0 liter of 50 mM ammonium acetate buffer, pH 6.0. The ZZ-tagged-IGF-1/IGF-1 mixture was lyophilized yielding a tan powder. After resuspension of the powder in a small volume (0.5 ml) of distilled water, the mixture was separated by polyacrylamide gel electrophoresis on a 15% acrylamide gel in a BioRad minigel electrophoresis apparatus for 1 hr, at 100 Volts. The protein bands (2) representing IGF-1 and ZZ-tagged-IGF-1, were stained by immersion of the gel in "gelcode" (Pierce), bromophenol blue stain for 2 hr. The gel was destained in distilled water prior to photographic documentation.

To remove the contaminating ZZ ligand and uncleaved ZZ-IGF-1 from the IGF-1 molecules, the cleavage mixture was passed over a small (3.0 ml) IgG-sepharose column previously washed with 10× vol of TST, 2× vol of TS and 2× vol of 5 mM ammonium acetate, pH 4.5. IGF-1 molecules passed through the column following the void volume (1.0 ml, determined by the elution of blue dextran), while the free ZZ domains and uncleaved ZZ-IGF bound to the IgG -sepharose. After collection of the column eluate (1.0 ml fractions), the amount of IGF-1 protein was measured by Bradford protein assay and the protein diluted with sterile water, divided into aliquots and frozen at -20°C for use in biological assays or structural studies.

### Cell Proliferation Assays

The biological activity of partially purified human IGF-1 preparations isolated from tobacco chloroplasts was established by measurement of IGF-1 effects on mammalian cell proliferation. Briefly, human megakaryoblastic (HU-3) cells carrying the IGF-1 receptor were cultured in RPMI-1640 medium + 10% calf serum. Cells in the logarithmic phase of growth were seeded into 96-well plates at a concentration of 25,000 cells/well in 50 μL RPMI-1640 containing 0.1 BSA and 0.5–1% calf serum. At the beginning of the assay, commercial human IGF-1 (Sigma), or IGF-1 partially purified from tobacco chloroplasts (50 μL) was added to each well at concentrations from 0.001 μg/ml to 10 μg/ml. The cell cultures were incubated at 37°C for 48 hours and a portion of each culture was stained with Trypan Blue dye to quantify cell viability prior to measurements of cell proliferation as determined most reliably by cell counting by hemocytometer.

Murine 3T3 fibroblasts also carrying the IGF-1 receptor were maintained in DMEM medium and seeded in 96-well plates from 2,000 cells/well to concentrations as high as 20,000 cells/well in 50 μL DMEM + 0.1% BSA containing 0.1–1% calf serum. IGF-1 (50 μL) was added in ascending concentrations from 0.001 to 10 μg/ml. Forty eight hours later (some time after 7 days) cell proliferation was measured by the CyQUANT NF Cell proliferation assay (Invitrogen Inc., San Diego, CA) and by hemocytometer counting. Proliferation was measured by fluorescence determination of DNA replication based on intercalation of a fluorescent dye into double stranded DNA. Cell proliferation was determined according to the instructions supplied by the manufacturer. Red, blue and green lines represent the best fit of the respective data points for each proliferation assay treatment. The commercial human IGF-1 used in the assay was Sigma-Aldrich Cat#3769 **(**10 μg-0.001 μg/ml).

## Authors' contributions

HD conceived and designed this study, interpreted data, wrote and revised several versions of this manuscript and obtained NIH/USDA funding to conduct this project. GR constructed chloroplast vectors, created and characterized transplastomic lines and wrote several sections of this manuscript. WL purified IGF-1 from transplastomic lines and contributed to manuscript sections on purification and functional evaluation. LS conducted mass spectrometry analysis and BD evaluated IGF-1 function in human cells in culture. All authors read and approved the final version of manuscript.
